# Long‐term prediction of epilepsy following traumatic brain injury among veterans using routine clinical data

**DOI:** 10.1002/epi.70430

**Published:** 2026-08-04

**Authors:** Mustafa Ozmen, Shashank Vadlamani, James J. Gugger, Megan Amuan, Ramon Diaz‐Arrastia, Mary Jo Pugh, Eamonn Kennedy

**Affiliations:** ^1^ Division of Epidemiology University of Utah Salt Lake City Utah USA; ^2^ Department of Electrical and Electronics Engineering Antalya Bilim University Antalya Turkey; ^3^ VA Salt Lake City Health Care System Informatics, Decision‐Enhancement, and Analytic Sciences Center Salt Lake City Utah USA; ^4^ Department of Neurology University of Rochester Rochester New York USA; ^5^ Department of Neurology University of Pennsylvania Perelman School of Medicine Philadelphia Pennsylvania USA

**Keywords:** epilepsy, posttraumatic epilepsy, prediction, traumatic brain injury, veteran

## Abstract

**Objective:**

Despite elevated risk for epilepsy following traumatic brain injury (TBI), there are limited tools to assess epilepsy risk following TBI using routine clinical data. The objective of this study was to develop and validate a machine learning approach to predict the onset of posttraumatic epilepsy (PTE) over varying time horizons following TBI, using only routine clinical data collected up to the month of TBI documentation.

**Methods:**

This retrospective longitudinal cohort study included post‐9/11 US veterans with a TBI diagnosis between 2008 and 2017 in US Department of War and Veterans Health Administration records. Machine learning models predicted PTE onset at 2, 5, and 10 years after the date of first TBI documentation. Only preinjury information was used for prediction. Model performance was evaluated on held‐out test data (30%). Shapley additive explanations (SHAP) were used to quantify the contribution of predictive features.

**Results:**

The cohort of 107 987 US veterans with TBI included 4930 (4.6%) incident epilepsy cases. Predicting epilepsy status, an optimized random forest model achieved area under curve [95% confidence interval] scores from receiver operating characteristic curves of .75 [.73–.77], .74 [.72–.75], and .73 [.72–.74] for 2‐, 5‐, and 10‐year forecast windows postinjury, respectively. High‐risk stratification identified 17.5% of all 5‐year epilepsy cases at a false positive rate of just 2.3% in the TBI population. SHAP analysis identified TBI severity and a cumulative preinjury comorbidity index as important predictors of PTE risk.

**Significance:**

Machine learning algorithms applied to routinely collected administrative health data can effectively stratify long‐term risk for epilepsy following TBI over a wide range of time horizons. These models open the possibility for enrichment of PTE cases in future preventative clinical trials using widely available routine clinical data.

**Key points**

- Machine Learning models using only routinely collected health data in a large veteran cohort accurately predicted epilepsy up to 10 years prior to first diagnosis.
- Epilepsy onset was predictable using only information up to time of TBI documentation at AUC = .73–.75 over 2‐, 5‐, and 10‐year time horizons postinjury.
- Percentile‐based epilepsy risk stratification models can enable significant cohort enrichment for future disease‐modifying clinical trials.

## 1 INTRODUCTION

Traumatic brain injury (TBI) is associated with increased risk for epilepsy.^1^ Epilepsy following TBI is a serious health concern that exacerbates chronic burdens for TBI patients and increases risk for heterogeneous adverse outcomes following TBI, including depression, headache, memory problems, and behavioral changes.^2^ Moreover, posttraumatic epilepsy (PTE) is a particular concern among post‐9/11 US veterans, who experience elevated rates of multiple related health comorbidities that may convey additional risk for epilepsy following TBI.^3^ Enhancing understanding and prediction of the risk for epilepsy in the years following TBI could help improve diagnostics, preventative treatments, and pathophysiological understandings, but there is often a latent period of many years between the initial TBI and epilepsy emergence.^1^ Although complications may arise during this time such as chronic increased inflammation, this latent period also presents a potential therapeutic window for future antiepileptogenic interventions.^4^ Prior work has investigated risk models and machine learning methods for clinical forecasting of a variety of high‐priority medical concerns, including seizure onset, drug‐resistant epilepsy, asthma, and homelessness.^5^, ^6^, ^7^, ^8^ However, current limitations in the ability to accurately identify those at greatest risk for epilepsy following TBI is a concern for the efficiency of potential clinical trials.^9^

TBI severity is a key indicator of epilepsy risk, but TBI is also associated with a spectrum of comorbidities such as stroke and mental health conditions that share bidirectional relationships with epilepsy.^10^, ^11^ Cognitive symptoms often precede diagnosis, suggesting that cognitive dysfunction may signal latent epileptogenesis.^12^ Integrating numerous diverse risk factors into predictive models is essential for robust modeling of PTE.

Although electroencephalography (EEG) and neuroimaging have predictive utility for risk of seizures, these modalities are resource‐intensive and often unavailable for large‐scale population screening.^13^ To address these limitations, machine learning approaches that use routine longitudinal electronic health records may offer a means to screen for epilepsy‐related outcomes without significant burden or intensive additional data collection.

However, several technical hurdles must be overcome to realize this goal. First, epilepsy is a challenging and heterogeneous diagnosis.^14^ To address this diagnostic complexity, this study employed an epilepsy outcome identification algorithm with a validated positive predictive value of .91–.94. Another concern is that simple risk models, including those that singlehandedly rely on comorbidity counts or stratification based on age or TBI severity, may not adequately capture interactions between overlapping comorbidities that convey risk for epilepsy. To address this, appropriate machine learning algorithms may have potential, because recent advances in biomedical forecasting of disease have significantly outperformed traditional approaches.^7^, ^8^, ^15^, ^16^, ^17^ This study assessed a variety of machine learning pipelines leveraging injury and preinjury health data for rapid epilepsy risk stratification at time of injury.

## 2 MATERIALS AND METHODS

### 2.1 Cohort description

A flow diagram describing the study inclusion and exclusion criteria is shown in Figure 1. The parent cohort included *N* = 910 951 post‐9/11 veterans with health records in the Veteran Health Administration (VHA) between 1999 and 2023. Veterans with history of TBI in the study period were included. Veterans with history of TBI prior to the study period were excluded. To allow for sufficient observation time after the first TBI diagnosis date (index date), only veterans with a first TBI diagnosis between the years 2008 and 2017 were included. To improve diagnostic clarity, we excluded veterans who had a service‐connected disability related to epilepsy with no evidence of seizure in medical records. We also excluded veterans with no epilepsy diagnosis who had evidence of seizures. Within the remaining cohort, we randomly held out 98 631 veterans, whom we are not using in this study, for a future prospective assessment of model performance. Finally, veterans with epilepsy diagnosis date prior to first TBI, or >10 years after first TBI, were excluded to ensure adequate data availability. The remaining cohort consisted of 107 987 veterans with TBI, 4930 of whom had an epilepsy diagnosis within 10 years of first TBI date.

**FIGURE 1:**
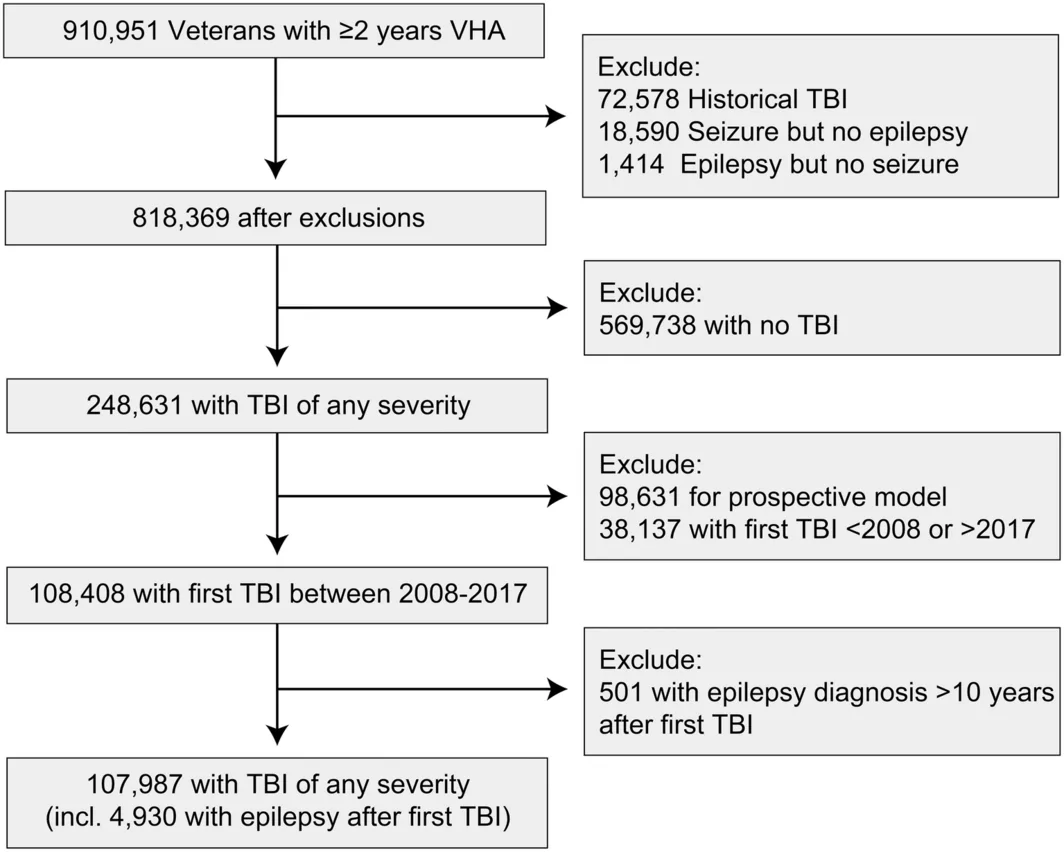
Cohort flow diagram. TBI, traumatic brain injury; VHA, Veteran Health Administration.

### 2.2 Epilepsy classification

To identify epilepsy, we carried out a previously validated epilepsy identification algorithm designed for use with VHA data.^18^ The validity of the algorithm in this population was measured by conducting chart abstraction of all new onset epilepsy patients from the South Texas Veterans Health Care System using a hierarchical approach to consider medication in the algorithm. In this cohort, an update to the epilepsy classification required the presence of certain key markers considering both International Classification of Diseases, 9th Revision (ICD‐9) and ICD‐10 codes, including diagnosis code(s) indicative of epilepsy (345 or G40) or convulsion (780.39 or R56) combined with documented use of antiseizure medication (ASM).^19^, ^20^ For ASMs, veterans treated exclusively with gabapentinoids were included only if they had an epilepsy‐specific diagnostic code in records. Relative to the clinical gold standard for epilepsy diagnosis, this algorithm previously achieved a positive predictive value (PPV) average of .91–.94 using chart review as reference standard.^21^

### 2.3 TBI classification

To classify TBI, the primary exposure, we used established diagnostic criteria for TBI classification according to Department of War (DoW) definitions in the US Military Health System.^22^ We note that recent literature has raised concerns regarding conventional TBI severity classification and nomenclature, but in this paper we used longstanding severity criteria including mild, moderate/severe, and penetrating TBI.^23^ Evidence of TBI in health records was drawn from multiple sources, including the DoW Trauma Registry, VHA inpatient and outpatient data, and TBI severity determined from the US Department of Veterans Affairs Comprehensive TBI Evaluation (CTBIE).^24^ TBI severity was determined based on the duration of alteration of consciousness or mental state, loss of consciousness (LOC), and posttraumatic amnesia, with information aggregated from both ICD‐9/10 codes and the CTBIE clinical interview according to established thresholds (i.e., mild TBI LOC < 30 min). When veterans met criteria for more than one TBI severity, the highest severity level was assigned. Classification of no TBI required no evidence of a classified or unclassified TBI.

### 2.4 Predictive features for modeling

To prospectively predict epilepsy, the model considered a wide array of features, including demographic and military measures, comorbidities, TBI characteristics and severity, and derived measures. Selection of comorbidities was based on their prevalence within the veteran community and their established associations with TBI and epilepsy based on expert consensus.^10^ The purpose of the study was to develop a model to prospectively validate risk for epilepsy at time of first TBI. Subsequent information such as mortality or repeated injuries after month of TBI would not be available at the time of epilepsy risk prediction and was not included. We defined 75 diagnostic variables according to the presence of groups of related ICD‐9/10 codes across DoW/VHA data, and other clinical records. These variables encompassed objective measures, disease diagnosis, and symptom‐based diagnoses, such as diabetes, headache, and alcohol abuse. A complete list of all comorbidities and their prevalences is provided in Table S1. However, many important variables such as lesion characteristics were not included in analysis, to keep the model dependent on routinely available clinical information for prospective population screening.

Demographic data and military characteristics were obtained from combined DoW/VHA administrative data. Military descriptive variables included most recent military components, rank, time since military separation, service‐connected disability, and deployment history. Individuals with more than one recorded date of deployment were classified as having multiple deployments.

### 2.5 Statistical analysis and synthetic minority oversampling techniques method

Demographic, military, and diagnostic data were combined to create a predictive dataset drawn from routinely collected and easily obtainable clinical information. As a primary outcome, epilepsy was rare overall, which leads to a class imbalance for prediction analysis where the proportion of negative class labels far outweighs the proportion of positive labels. To ensure good representation and improve model performance, we implemented synthetic minority oversampling techniques (SMOTE) after splitting the training and test data portions on only the training data by adding synthetic observations on the minority class (epilepsy) and randomly undersampling the majority class (no epilepsy) on the raw training data.^25^ After applying SMOTE to the training data, the epilepsy group accounted for approximately 25% of subjects. Model performance was evaluated using a separate unmodified sample of veterans for testing and validation with no SMOTE synthetic data used in testing.

### 2.6 Survival analysis

To assess the emergence of epilepsy following TBI over time, Kaplan–Meier curves were developed using the plotlines survival analysis package.^26^ Time zero was defined as the date of the first TBI documentation. To assign end dates, the earliest occurrence of the first epilepsy diagnosis, death, or the end of the study period in 2025 was used, with veterans censored accordingly. Conversely, inclusion of future information about death is not appropriate in a forecasting model due to future data leakage concerns.

### 2.7 Detection

Various detection schemes were evaluated using multiple post‐TBI time frames to assess temporal sensitivity to epilepsy detection. We selected three different time frames (0–2, 0–5, 0–10 years following TBI) to investigate how the ability to predict epilepsy after TBI varied with postinjury follow‐up duration. For each time frame, a separate binary outcome and cohort were defined. For example, in the model predicting epilepsy within 5 years of the TBI date, veterans with a first epilepsy diagnosis >5 years after the TBI date were excluded from that specific analysis.

For each model training and evaluation, the cohort was randomly split into 70% training and 30% test groups. To ensure reproducibility and reduce stochastic variability, all random train‐test splitting, randomization, and stochastic model processes were initialized using a fixed random seed.

### 2.8 Model selection

Three methods were applied: a logistic regression model, a random forest classifier, and an ensemble approach that combined multiple random forest and gradient‐boosting models with different parameter settings. Logistic regression estimated epilepsy risk using a linear combination of input features through a logistic (sigmoid) function to estimate the probability of epilepsy onset.

Although penalized regression approaches such as least absolute shrinkage and selection operator can also be used for PTE prediction, as described by Awan et al.,^5^ we selected tree‐based ensemble methods for several reasons. First, random forest models can intrinsically tolerate collinearity through a process where each individual tree in the forest sees only a small number of the total features provided to the model. Second, our dataset contains 75 sparse diagnostic variables where nonlinear interactions are well captured by random forest without manual feature engineering. These were strengths that benefitted model performance.

The random forest classifier consisted of 1000 decision trees, each trained on a bootstrap sample of the variables available. All probabilities across trees for each veteran were then aggregated to produce a single decision for each veteran. The third model, an ensemble, combined three random forest and two gradient boosting methods each with grid‐search optimized parameters. A positive prediction was made when at least three of the five ensemble models produced a predicted epilepsy risk exceeding 95% probability.

To improve explainability, Shapley additive explanations (SHAP) values were used to quantify the contribution of individual features at observation level in the machine learning models.^27^ SHAP values estimate the average marginal contribution of each feature. Model discrimination was evaluated using receiver operating characteristic (ROC) curves, and performance was summarized by the accuracy, PPV, and true and false positive rates.

### 2.9 Standard protocol approvals, registrations, and patient consent

The study was reviewed and approved by the University of Utah's institutional review board with a waiver of informed consent. This study was limited to the secondary use of deidentified data and was determined to be nonhuman subjects research.

## 3 RESULTS

After exclusion criteria, the cohort was comprised of *n* = 107 987 veterans with a documented history of TBI. Demographic, military, and injury characteristics are shown in Table 1 for the full cohort, and also for the epilepsy‐positive and ‐negative groups separately. The mean age at the index date was 30.6 years, and 7.3% of the veterans were female. All veterans had a documented history of TBI, 67.5% of whom had mild TBI severity, 14.0% had moderate/severe TBI severity, and 2.6% had evidence of penetrating TBI (the remainder had TBI of unclassified severity). Demographic and military measures were broadly similar across the epilepsy status groups. However, higher severity TBI was observed more frequently in the epilepsy‐positive group.

**TABLE 1:** Demographic and military characteristics of the cohort.

Characteristic	All	No EPI	EPI
Sample size, *n*	107 987	103 057	4930
Age at index, years			
Mean (SD)	30.6 (9.0)	30.6 (9.0)	30.6 (8.9)
Sex			
Male	92.7%	92.8%	91.4%
Female	7.3%	7.2%	8.6%
Race			
Black	14.1%	14.3%	11.5%
White	77.3%	77.1%	81.9%
Other	8.6%	8.7%	6.6%
Ethnicity			
Hispanic/Latino	14.9%	14.9%	13.7%
Employment			
Unemployed	49.3%	48.9%	58.3%
Geography			
Urban	65.4%	65.5%	62.3%
Married/partnered			
Yes	50.4%	50.4%	51.2%
Military			
Active duty	69.3%	69.2%	71.2%
Reserve/National Guard	30.7%	30.8%	28.8%
Enlisted	95.5%	95.5%	96.0%
Officer/WO	4.5%	4.5%	4.0%
Time since separation, years	14.7	14.7	14.7
Multiple deployments	55.0%	55.3%	50.2%
SCD < 10%	6.8%	7.0%	2.3%
SCD = 10%–50%	8.3%	8.6%	3.5%
SCD = 50%+	84.9%	84.4%	94.2%
TBI Severity			
Mild	67.5%	67.9%	59.5%
Moderate/severe	14.0%	13.6%	22.2%
Penetrating	2.6%	2.3%	7.0%
Unclassified	15.9%	16.2%	11.3%

Abbreviations: EPI, epilepsy; SCD, Service‐Connected Disability; TBI, traumatic brain injury; WO, warrant officer.

Figure 2 illustrates the Kaplan–Meier curves of cumulative incidence of epilepsy in the cohort following the date of first documented TBI, stratified by TBI severity. The number of veterans at risk in each TBI severity group is tabulated below, following the TBI index date. The survival curves for mild (blue), moderate/severe (orange), and penetrating (green) TBI indicate progressively increasing risk of epilepsy over time with higher severity injuries. Veterans with unclassified TBI exhibited a similar, overlapping trajectory to those with mild TBI (data not shown).

**FIGURE 2:**
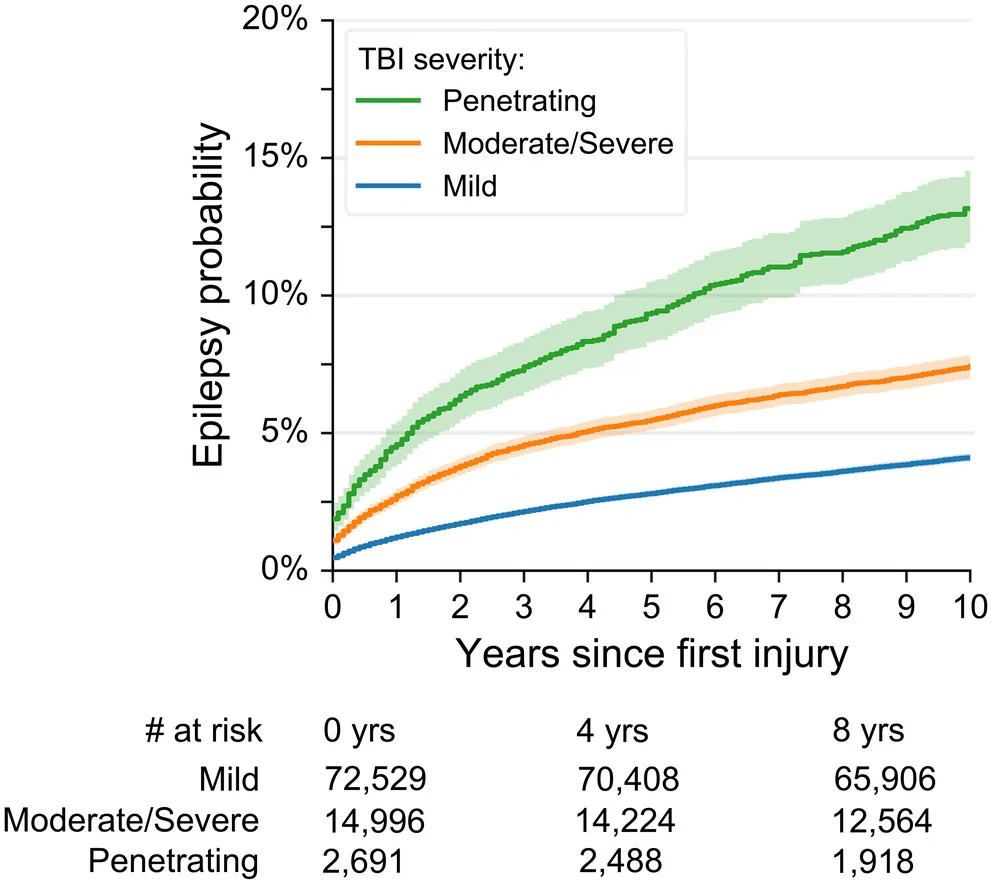
Kaplan–Meier curves illustrating risk of epilepsy broken out by traumatic brain injury (TBI) severity: mild (blue), moderate/severe (orange), penetrating (green).

Figure 3 compares the performance of the three prediction models on test data. The ROC curves of logistic regression, random forest, and ensemble models are shown. Figure 3A illustrates results for the 2‐year follow‐up window model, summarized by area under the curve (AUC; 95% confidence interval [CI]) for logistic regression of .65 (95% CI = .63–.67), for random forest of .75 (95% CI = .73–.77), and for the ensemble model of .72 (95% CI = .70–.74). Figure 3B demonstrates a very similar trend for the 5‐year follow‐up window, with the random forest model consistently outperforming the other two approaches. Figure 3C displays results for the 10‐year prediction window, in which the tree‐based machine learning approaches did not substantially outperform logistic regression; however, all models still performed adequately (logistic regression: .71 [95% CI = .70–.73], random forest: .73 [95% CI = .72–.74], ensemble: .72 [95% CI = .71–.74]). Overall, the random forest model was optimal averaged across scenarios.

**FIGURE 3:**
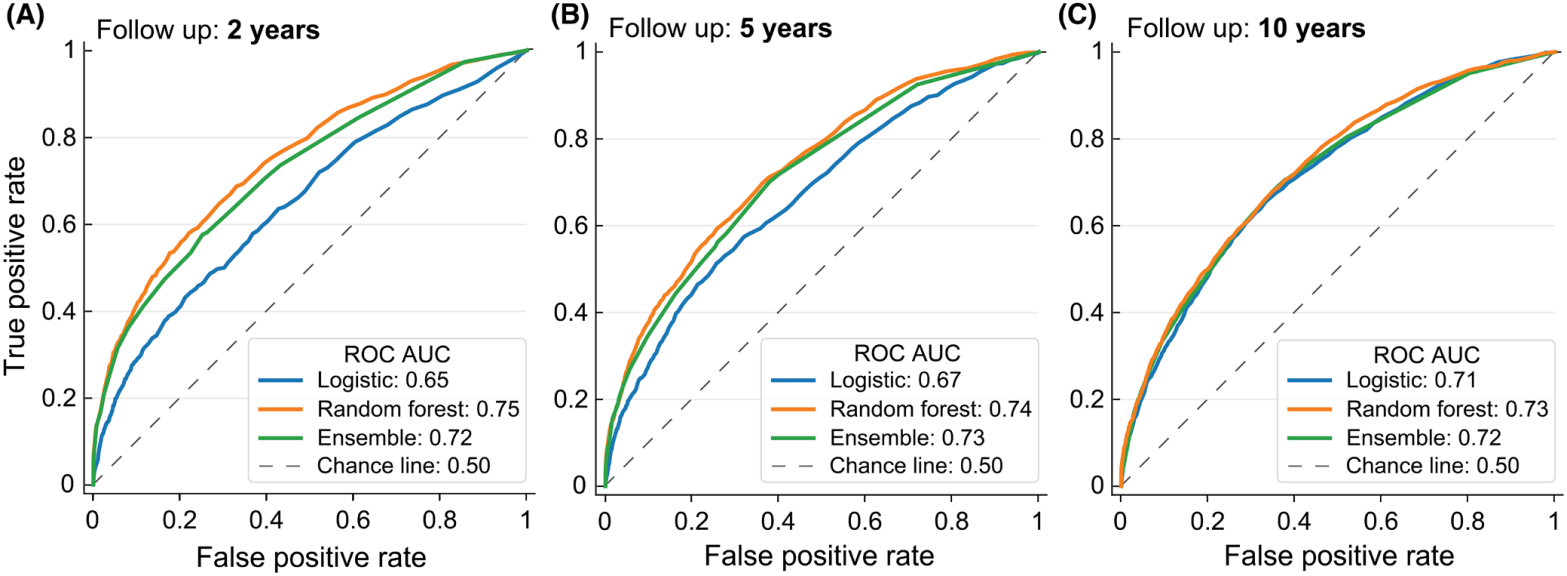
Prediction performances for 2, 5, and 10 years following up first TBI by logistic regression, random forest, and ensemble methods. AUC, area under curve; ROC, receiver operator characteristic.

Results for the random forest model are presented in Table 2 across all three time frames, stratified by different percentile‐based risk thresholds that were used to identify epilepsy‐positive and ‐negative cases. A clear trade‐off was observed between improved model sensitivity and lower false positive rates as percentile thresholds increased. The risk models were determined by the training data (70% of the cohort) only, and these thresholds were applied to the independent test data to generate performance estimates. Prespecified percentile thresholds (1%–20%) were used to evaluate veterans at elevated risk of epilepsy (Table 2).

**TABLE 2:** Posttraumatic epilepsy prediction model performance results on test data shown across varying percentile‐based detection thresholds.

Risk model	Percentile threshold	Identified at risk, *n*	SN, % of all cases	FPR	PPV	Accuracy
Two‐year follow up, *n* = 105 313 (2256 epilepsy cases)						
1	1%	64	6.9%	.001	.734	.980
2	2%	272	13.0%	.006	.324	.976
3	3%	605	17.0%	.016	.190	.967
4	4%	990	22.7%	.027	.156	.957
5	5%	1371	27.6%	.038	.136	.947
6	10%	3345	41.2%	.099	.083	.890
7	15%	5239	50.7%	.158	.065	.834
8	20%	7152	57.8%	.219	.055	.777
Five‐year follow up, *n* = 106 579 (3522 epilepsy cases)						
1	1%	75	4.9%	.001	.693	.968
2	2%	269	9.6%	.005	.379	.965
3	3%	537	14.2%	.013	.279	.960
4	4%	891	17.5%	.023	.208	.951
5	5%	1212	20.1%	.032	.175	.942
6	10%	3192	36.4%	.091	.121	.891
7	15%	5109	45.1%	.150	.093	.837
8	20%	7030	54.0%	.209	.081	.783
Ten‐year follow up, *n* = 107 987 (4930 epilepsy cases)						
1	1%	75	4.3%	.000	.853	.956
2	2%	215	6.8%	.004	.465	.954
3	3%	467	10.3%	.010	.328	.949
4	4%	768	13.6%	.018	.262	.943
5	5%	1102	16.1%	.028	.216	.935
6	10%	2953	31.0%	.081	.155	.891
7	15%	4948	42.2%	.140	.126	.840
8	20%	6856	49.8%	.198	.107	.788

*Note*: Models predicted epilepsy onset at 2‐, 5‐, and 10‐year follow‐up after TBI using only preinjury information. Performance was similar across follow‐up time windows. Percentile‐based detection thresholds were determined from training data and applied to the test data. Model performance metrics are demonstrated for each risk model and follow‐up interval. Equivalent tabulated results are shown in Table S2 for logistic regression.

Abbreviations: FPR, false positive rate; PPV, positive predictive value; SN, sensitivity.

For each risk model (threshold), the sensitivity (detection), false‐positive rate, PPV, and accuracy are reported. At 2‐year follow‐up, risk model 8 correctly identified 57.8% of all individuals who subsequently developed epilepsy in the TBI cohort, whereas risk model 2 identified just 13.0% of epilepsy cases, but with a substantially low false positive rate of .006 among veterans who did not develop epilepsy (see bold table text). At 5 years postinjury, model 4 identified 17.5% of all 5‐year epilepsy cases at a false positive rate of just 2.3% in the TBI population (see bold table text). Note that the analysis was conducted separately for 2‐, 5‐, and 10‐year prediction windows; therefore, the underlying population differs slightly by time frame. As the time since injury increased to 10 years, the model's performance declined modestly, perhaps due to the lower probability of a true biological link between TBI and the emergence of epilepsy. Overall, these results suggest significant improvements in the efficiency of identifying epilepsy cases after TBI across a wide range of time frames since injury.

To explore feature importance for epilepsy prediction, Figure 4 displays SHAP values derived from the 5‐year random forest model. The most influential covariates are grouped into categories representing TBI severity, summary measures, neurological diagnoses, substance abuse, and psychiatric diagnoses. Similar results were observed across time frames. The comorbidity index is a summary measure derived using all available comorbidity data spanning 3 years prior to and including the month of the TBI index date. The details regarding derivation of comorbidity index are provided in Appendix S1.

**FIGURE 4:**
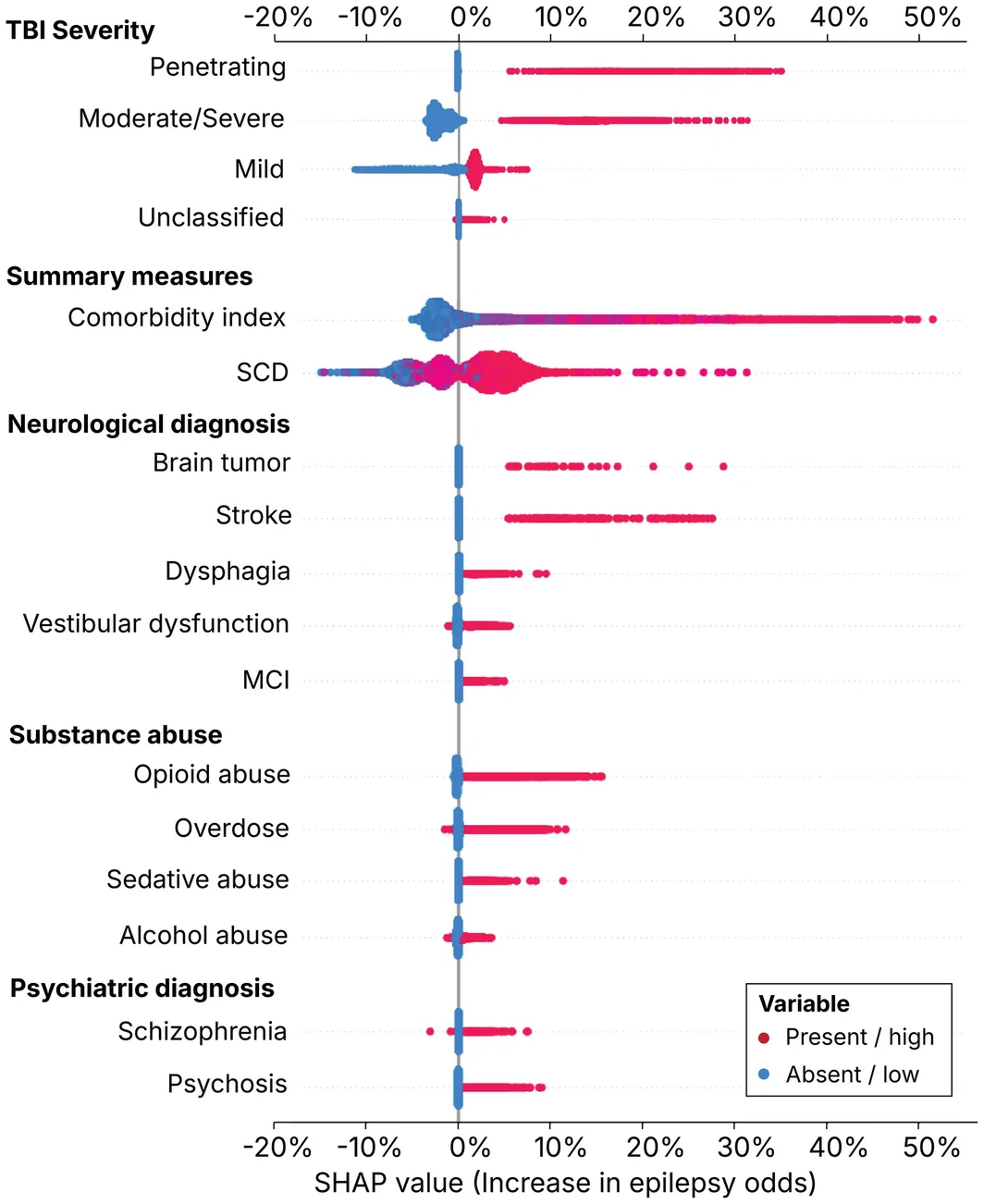
Shapley additive explanations (SHAP) values are shown from the random forest model. A comorbidity index of conditions prior to the time of injury was a high‐utility feature. MCI, mild cognitive impairment; SCD, service‐connected disability; TBI, traumatic brain injury.

## 4 DISCUSSION

PTE arises from a complex interaction of prior genetic, neurological, and psychological risk factors that interact and accumulate over an individual's lifetime and are further modified by TBI. The goal of this study was to forecast the risk of PTE development among veterans using only routinely collected clinical data available in VHA systems as part of standard treatment and diagnostics. Accurate early prognostic detection of individuals at high risk for PTE could provide a means to test the evaluation of future disease‐modifying interventions aimed at preventing epilepsy, potentially years prior to seizure onset. Although there are as yet no known therapies that effectively prevent the development of PTE,^28^ improving early detection remains a critical prerequisite for the development and testing of future antiepileptogenic or disease‐modifying therapies.

Because the clinical utility of prediction varies across different time frames, in this study, we evaluated the model performance at 2‐year, 5‐year, and 10‐year intervals to forecast veterans' PTE risk following the initial TBI. Across all performance metrics, the random forest model had the strongest overall predictive performance. In the 2‐year follow‐up model, classifying high‐risk veterans as those within the top 3% estimated cumulative epilepsy risk identified 17% epilepsy cases in the TBI cohort, with a false positive rate of only 1.6%. This represents a significant enrichment compared with current estimates of numbers needed to treat and prevent epilepsy.^19^ Comparable performances were observed for the 5‐year prediction window, where a top 4% risk level to forecast epilepsy onset yielded 17.5% sensitivity at 2.3% false positive rate. Predictive performance declined modestly for the 10‐year postinjury forecasting. This may be tentatively attributed to the loss of some causal relationship between TBI risk for epilepsy over very long‐time frames and increasing possibility of mediating factors postinjury (5 percentile risk model at +10 years: 16.1% sensitivity and 2.8% false positive rate).^29^

Using balanced AUC as a summary metric, the random forest model achieved values of .75, .74, and .73, at 2, 5, and 10 years, respectively. Surprisingly, these results are comparable to studies that leverage EEG and magnetic resonance imaging (MRI) data for individual‐level prediction of epilepsy onset, such as Akrami et al. (AUC = .78).^13^ We note that the current study sample size is several orders of magnitude higher than these prior reports. In contrast to imaging‐ and electrophysiology‐based approaches, our method is scalable to entire health care systems using routine clinical system data. In this paper, the approach has already been applied to >100 000 patients with records of TBI in the VHA data repository.

Our findings were consistent with the hypothesis that machine learning approaches would outperform the predictive performance of conventional regression models. Beyond enhanced performance, the use of SHAP values enabled transparent interpretation of model processes. SHAP analysis identified TBI severity, cumulative count of comorbidities, neurological diagnosis, substance misuse (particularly opioids; Figure 4), and psychiatric comorbidities as key contributors to PTE risk. These findings align with existing literature, where one of the most robust clinical predictors for PTE is the severity of the initial brain injury.^30^ Beyond severity, specific clinical risk factors amplify the PTE risk, including lesion types (e.g., temporal or frontal contusions, subdural hematoma) and the presence of early posttraumatic seizures within 7 days of injury.

According to guidance from the US Food and Drug Administration, achieving high sensitivity and specificity for identifying individuals for clinical enrichment studies is encouraged.^31^ This study provides interesting insights into the trade‐offs inherent in clinical detection models. With our models, a screening cutoff can be applied shortly after TBI to identify high‐risk patients who may be suitable for clinical studies. As increasing sensitivity is associated with a higher false positive rate, the risk thresholds may serve as a practical guide for clinicians. However, there are several barriers that prevent the immediate clinical translation of the proposed model. Currently available ASMs are not known to prevent PTE, but identifying high‐risk individuals using data collected as part of routine clinical practice would facilitate enrichment of clinical trials of disease‐modifying treatments.^32^ Our results suggest that model performance was relatively stable across prediction windows, which supports flexibility and relevance to a wide range of potential trial designs. Focusing initially on clearly defined high‐risk groups may maximize the likelihood of detecting effective preventative clinical trials.

Finally, this study provides a useful foundation for low‐cost, large‐scale screening using existing health care data. Model performance could be further enhanced by incorporating additional steps such as MRI, patient‐reported measures, or biomarkers for targeted individuals identified as high risk. Future work should assess whether these additions improve predictive accuracy and should include prospective validation in independent cohorts.

### 4.1 Limitations

When interpreting the findings in this study, there are several limitations that should be considered. First, our models exclusively rely on routinely collected administrative and clinical data from the VHA. These data may have coding inaccuracies, underreporting, and temporal inaccuracies in ICD diagnoses. Because the forecasting model cannot utilize future information such as death without losing prospective utility, our primary approach treats all participants as at risk at baseline. Second, although the cohort size was large, the main outcome was relatively rare, which may result in limited sensitivity despite high accuracy. Third, the decline in prediction performance for longer time frames suggests that the association between TBI and epilepsy reduced over time, or the influence of unmeasured postinjury factors influences the TBI and epilepsy relationship. Fourth, in post hoc analysis, adding pharmaceutical data did not significantly affect detection performance. However, medications were used for classification in the epilepsy algorithm. ASMs prescribed in veterans for nonepilepsy conditions (chronic pain, neuropathic pain, migraine, mood stabilizations) may introduce error, and our algorithm applied specific conditions on veterans prescribed gabapentinoids but not other ASMs. We elected to develop our models using routinely documented clinical information, but further extensions of the PTE prediction model could glean more granular and acute measures such as surgical decisions given intracranial hemorrhage to improve predictive performance.

However, the primary objective of this study was to predict epilepsy following TBI, and our prospective framing does not distinguish between prediction of epilepsy that resulted directly from a TBI and risk for epilepsy after TBI conveyed through other intermediary mechanisms and conditions, such as intermediary stroke after TBI. The index date was defined as date of first TBI, and so we also did not consider the potential cumulative impacts of multiple or repeated injuries after our observation time window. Multiple injuries have been associated with chronic psychological consequences following mild TBI in military populations.^33^

## 5 CONCLUSIONS

This study of veterans with history of TBI found that machine learning algorithms applied to routinely collected administrative health data prior to time of injury can effectively stratify long‐term risk for epilepsy following TBI over a wide range of time horizons. Thus, this study suggests a path for effective enrichment of prognostic PTE cases in future preventative clinical trials, provided that good model performance persists in prospective datasets.

## AUTHOR CONTRIBUTIONS

**Mustafa Ozmen:** Data curation; formal analysis; methodology; software; validation; visualization; writing—original draft preparation; writing—review and editing. **Shashank Vadlamani:** Data curation; formal analysis; investigation; software; writing—original draft preparation; writing—review and editing. **James J. Gugger:** Writing—original draft preparation; writing—review and editing. **Megan Amuan:** Data curation; writing—original draft preparation; writing—review and editing. **Ramon Diaz‐Arrastia:** Writing—review and editing. **Mary Jo Pugh:** Conceptualization; resources; writing—original draft preparation; writing—review and editing. **Eamonn Kennedy:** Conceptualization; funding acquisition; investigation; methodology; resources; supervision; visualization; writing—original draft preparation; writing—review and editing.

## FUNDING INFORMATION

This study was funded by the US Department of War Epilepsy Research Program grant EP220089 (award no. HT9425‐23‐1‐0221).

## CONFLICT OF INTEREST STATEMENT

The authors declare no conflict of interest. We confirm that we have read the Journal's position on issues involved in ethical publication and affirm that this report is consistent with those guidelines.

## Supporting information


**Appendix S1.** Comorbidity index (utility score) calculation.

**Table S1.** Table of categories and individual variables used for prediction models, with all included comorbidities listed alphabetically.

**Table S2.** Posttraumatic epilepsy prediction model results on test data shown for logistic regression. Models were tasked to predict who would develop epilepsy at 2‐, 5‐, and 10‐year follow‐up after traumatic brain injury (TBI) using only preinjury information. Performance was similar across follow‐up time windows. Percentile detection thresholds were determined from training data. At 2‐year follow‐up from injury using risk model 8, 40.8% of all individuals who subsequently developed epilepsy in the TBI cohort were correctly identified. Conversely, risk model 2 at 2‐year follow‐up identified just 10.5% of epilepsy cases, but with a low false positive rate of .018 among CFE who did not develop epilepsy.

## Data Availability

Raw data are not available due to the inclusion of sensitive medical data and identifiable information. Summary deidentified statistics may be made available upon reasonable request, pending appropriate study approvals and data transfer agreements between participating institutions. The code used for analysis is available upon request.

## References

[1] Frey LC . Epidemiology of posttraumatic epilepsy: A critical review. Epilepsia. 2003;44:11–17. 10.1046/j.1528-1157.44.s10.4.x 14511389

[2] Pugh MJ , Kennedy E , Prager EM , Humpherys J , Dams‐O'Connor K , Hack D , et al. Phenotyping the Spectrum of traumatic brain injury: A review and pathway to standardization. J Neurotrauma. 2021;38(23):3222–3234. 10.1089/neu.2021.0059 PMC8917880 33858210

[3] Salazar AM , Jabbari B , Vance SC , Grafman J , Amin D , Dillon JD . Epilepsy after penetrating head injury. I. Clinical correlates: A report of the Vietnam head injury study. Neurology. 1985;35(10):1406–1414. 10.1212/wnl.35.10.1406 3929158

[4] Webster KM , Sun M , Crack P , O'Brien TJ , Shultz SR , Semple BD . Inflammation in epileptogenesis after traumatic brain injury. J Neuroinflammation. 2017;14(1):10. 10.1186/s12974-016-0786-1 PMC5237206 28086980

[5] Awan N , Kumar RG , Juengst SB , DiSanto D , Harrison‐Felix C , Dams‐O'Connor K , et al. Development of individualized risk assessment models for predicting post‐traumatic epilepsy 1 and 2 years after moderate‐to‐severe traumatic brain injury: A traumatic brain injury model system study. Epilepsia. 2025;66(2):482–498. 10.1111/epi.18210 PMC11827721 39655874

[6] Haneef Z , Eisenschenk S , Lopez MR , Hildebrand A , Rehman R , Gopinath Karicheri S , et al. Optimizing drug‐resistant epilepsy identification in the veterans health administration. Epilepsy Res. 2025;214:107568. 10.1016/j.eplepsyres.2025.107568 40300417

[7] Finkelstein J , Jeong IC . Machine learning approaches to personalize early prediction of asthma exacerbations. Ann N Y Acad Sci. 2017;1387(1):153–165. 10.1111/nyas.13218 PMC5266630 27627195

[8] Koh KA , Montgomery AE , O'Brien RW , Kennedy CJ , Luedtke A , Sampson NA , et al. Predicting homelessness among U.S. Army soldiers No longer on active duty. Am J Prev Med. 2022;63(1):13–23. 10.1016/j.amepre.2021.12.028 PMC9219110 35725125

[9] Karlander M , Håkansson S , Ljungqvist J , Sörbo A , Zelano J . Risk of epilepsy following a first posttraumatic seizure: A register‐based study. Neurol Clin Pract. 2025;15(1):e200409. 10.1212/CPJ.0000000000200409 PMC11588421 39600560

[10] Ozmen M , Vadlamani S , Gugger JJ , Amuan M , Cheney A , Diaz‐Arrastia R , et al. From acute injury to chronic comorbidity: Interrupted time series modeling of traumatic brain injury impact among post‐9/11 veterans. PLoS One. 2025;20(11):e0325411. 10.1371/journal.pone.0325411 PMC12591432 41196861

[11] Tritt A , Yue JK , Ferguson AR , Torres Espin A , Nelson LD , Yuh EL , et al. Data‐driven distillation and precision prognosis in traumatic brain injury with interpretable machine learning. Sci Rep. 2023;13(1):21200. 10.1038/s41598-023-48054-z PMC10692236 38040784

[12] Helmstaedter C , Witt JA . Epilepsy and cognition–A bidirectional relationship? Seizure. 2017;49:83–89. 10.1016/j.seizure.2017.02.017 28284559

[13] Akrami H , Cui W , Kim PE , Heck CN , Irimia A , Jerbi K , et al. Prediction of post traumatic epilepsy using MR‐based imaging markers. Hum Brain Mapp. 2024;45(17):e70075. 10.1002/hbm.70075 PMC11574740 39560185

[14] Beniczky S , Trinka E , Wirrell E , Abdulla F , al Baradie R , Alonso Vanegas M , et al. Updated classification of epileptic seizures: Position paper of the international league against epilepsy. Epilepsia. 2025;66:1804–1823. 10.1111/epi.18338 PMC12169392 40264351

[15] Pati S , Baid U , Edwards B , Sheller M , Wang SH , Reina GA , et al. Federated learning enables big data for rare cancer boundary detection. Nat Commun. 2022;13:7346. 10.1038/s41467-022-33407-5 PMC9722782 36470898

[16] Theodorou B , Xiao C , Glass L , Sun J . MediSim: Multi‐granular simulation for enriching longitudinal, multi‐modal electronic health records. Patterns. 2025;6(6):101261. 10.1016/j.patter.2025.101261 PMC12191717 40575129

[17] de Lacy N , Ramshaw M , Lam WY . RiskPath: Explainable deep learning for multistep biomedical prediction in longitudinal data. medRxiv. 2025;6:101240. 10.1016/j.patter.2025.101240 PMC12365533 40843347

[18] Pugh MJ , Van Cott AC , Cramer JA , Knoefel JE , Amuan ME , Tabares J , et al. Trends in antiepileptic drug prescribing for older patients with new‐onset epilepsy: 2000–2004. Neurology. 2008;70:2171–2178. 10.1212/01.wnl.0000313157.15089.e6 18505996

[19] Pease M , Mittal A , Merkaj S , Okonkwo DO , Gonzalez‐Martinez JA , Elmer J , et al. Early seizure prophylaxis in mild and moderate traumatic brain injury: A systematic review and meta‐analysis. JAMA Neurol. 2024;81(5):507–514. 10.1001/jamaneurol.2024.0689 PMC11002778 38587858

[20] Coelho LMG , Blacker D , Hsu J , Newhouse JP , Westover MB , Zafar SF , et al. Association of Early Seizure Prophylaxis with Posttraumatic Seizures and Mortality. Neurol Clin Pract. 2023;13(3):e0200145. 10.1212/CPJ.0000000000200145 PMC10101717 37066107

[21] Pugh MJV , Orman JA , Jaramillo CA , Salinsky MC , Eapen BC , Towne AR , et al. The prevalence of epilepsy and association with traumatic brain injury in veterans of the Afghanistan and Iraq wars. J Head Trauma Rehabil. 2015;30(1):29–37. 10.1097/HTR.0000000000000045 24695268

[22] Swanson TM , Isaacson BM , Cyborski CM , French LM , Tsao JW , Pasquina PF . Traumatic brain injury incidence, clinical overview, and policies in the US military health system since 2000. Public Health Rep. 2017;132(2):251–259. 10.1177/0033354916687748 PMC5349478 28135424

[23] Manley GT , Dams‐O'Connor K , Alosco ML , Alosco ML , Awwad HO , Bazarian JJ , et al. A new characterisation of acute traumatic brain injury: The NIH‐NINDS TBI classification and nomenclature initiative. Lancet Neurol. 2025;24(6):512–523. 10.1016/S1474-4422(25)00154-1 40409315

[24] Belanger HG , Powell‐Cope G , Spehar AM , CCRC , McCranie M , Klanchar SA , et al. The veterans health Administration's traumatic brain injury clinical reminder screen and evaluation: Practice patterns. J Rehabil Res Dev. 2016;53(6):767–780. 10.1682/JRRD.2015.09.0187 28273328

[25] Fernández A , García S , Herrera F , Chawla NV . SMOTE for learning from imbalanced data: Progress and challenges. Pattern Recognit. 2018;61:326–343.

[26] Davidson‐Pilon C . Lifelines: Survival analysis in python. J Open Source Softw. 2019;4(40):1317. 10.21105/joss.01317

[27] Lundberg SM , Lee SI . A unified approach to interpreting model predictions. Adv Neural Inf Proces Syst. 2017;30:4765–4774.

[28] Foresti ML , Garzon E , de Moraes MT , Valeriano RPS , Santiago JP , dos Santos GM , et al. Initial clinical evidence on biperiden as antiepileptogenic after traumatic brain injury–A randomized clinical trial. Front Neurol. 2024;15:1443982. 10.3389/fneur.2024.1443982 PMC11339878 39175759

[29] Vadlamani S , Ozmen M , Gugger JJ , Cheney A , Amuan M , Diaz‐Arrastia R , et al. Mediators of epilepsy risk after traumatic brain injury: A 20‐year U.S. veteran cohort study. Epilepsia. 2025;66(4):1177–1186. 10.1111/epi.18248 PMC11997920 39729030

[30] Mavroudis I , Franekova K , Petridis F , Ciobica A , Dăscălescu G , Anton CR , et al. Post‐traumatic epilepsy after mild and moderate traumatic brain injury: A narrative review and development of a clinical decision tool. Reports. 2025;8(4):193. 10.3390/reports8040193 PMC12643447 41133534

[31] U.S. Food and Drug Administration . Enrichment Strategies for Clinical Trials to Support Determination of Effectiveness of Human Drugs and Biological Products: Guidance for Industry. Silver Spring (MD): FDA; 2019.

[32] Karamian A , Farzaneh H , Taheri M , Seifi A . Effectiveness of levetiracetam versus phenytoin in preventing seizure in traumatic brain injury patients: A systematic review and meta‐analysis. Clin Neurol Neurosurg. 2024;240:108251. 10.1016/j.clineuro.2024.108251 38569246

[33] Kennedy E , Ozmen M , Bouldin ED , Panahi S , Mobasher H , Troyanskaya M , et al. Phenotyping depression after mild traumatic brain injury: Evaluating the impact of multiple injury, gender, and injury context. J Neurotrauma. 2024;41:924–933. 10.1089/neu.2023.0381 38117134

