# Supplementary material for: Long‐term prediction of epilepsy following traumatic brain injury among veterans using routine clinical data
Source: Epilepsia. 2026 Aug 4;67(11):5840–50. doi: 10.1002/epi.70430 (PMC13768913; doi:10.1002/epi.70430)
Supplement: Supplementary file 1 [file EPI-67-5840-s001.docx]

**Appendix S1: Comorbidity Index (Utility Score) calculation**

$$ci= \sum_{j=1}^{75} log\left( 1+\sum_{t=-36}^{0} u_{j, t} \right)$$

Let $u_{j,t}$denote the number of healthcare utilization events (including diagnostic encounters) attributable to comorbidity $j$at time $t$, where time is indexed as months relative to the TBI index date. In this formulation, diagnoses were aggregated for each comorbidity over the three years preceding the TBI index date. A μ-law–based logarithmic transformation was then applied to the utilization count of each comorbidity to compress high-frequency usage while preserving contributions from multiple distinct comorbidities. This transformation emphasizes breadth of comorbidity-related healthcare utilization over intensity within any single condition. For example, a diagnosis of 100 different conditions has more impact than 100 diagnosis of the same single condition, which is preferable because repeated coding per visit for the same concern is an often observed empirical trend in the health record. The final comorbidity index was computed as the sum of the transformed utilization values across all 75 comorbidities from t=-36 months before TBI to TBI index date.
