# Supplementary material for: Long‐term prediction of epilepsy following traumatic brain injury among veterans using routine clinical data
Source: Epilepsia. 2026 Aug 4;67(11):5840–50. doi: 10.1002/epi.70430 (PMC13768913; doi:10.1002/epi.70430)
Supplement: Supplementary file 2 [file EPI-67-5840-s002.docx]

| Category | Variables included |
| --- | --- |
| Sociodemographics | Age, Male sex, Hispanic ethnicity, White, Black, Asian American and Pacific Islander (AAPI), Native American, Unemployed, Married, Urban residence |
| Military Service Characteristics | Multiple deployments, Reserve/National Guard status, Enlisted rank, Service-connected disability (SCD), Military service start year, Military service end year, Honorable discharge |
| Military Occupation Specialties | Infantry, Special forces, Military training instructor, Armor/amphibious, Combat engineer, Artillery gunnery, Rocket artillery, Missile artillery |
| Traumatic Brain Injury (TBI) | TBI diagnosis, Year of TBI, Mild TBI, Moderate/severe TBI, Penetrating TBI, Unclassified TBI, Occupational blast risk |
| Clinical Conditions & Comorbidities (A–B) | Adjustment disorder, Alcohol abuse, Alzheimer's disease, Amphetamine abuse, Amyotrophic lateral sclerosis (ALS), Anoxic brain injury, Anxiety, Atherosclerosis, Attention deficit, Back pain, Bipolar disorder, Brain tumor, Fall history |
| Clinical Conditions & Comorbidities (C–E) | Cancer, Cannabis abuse, Cardiac disease, Chronic fatigue, Cirrhosis, Cocaine abuse, Constipation, Conversion disorder, Coronary artery disease (CAD), Dementia, Depression, Diabetes, Drug-induced mental disorder, Dysphagia, Encephalopathy |
| Clinical Conditions & Comorbidities (F–L) | Fatty liver, Frontotemporal dementia (FTD), Hallucinogen abuse, Headache, Hearing loss, Hepatitis C, Hypercholesterolemia, Hypersomnia, Hypertension, Injury, Insomnia, Irritable bowel syndrome (IBS), Kidney disease, Liver disease, Lung disease |
| Clinical Conditions & Comorbidities (M–O) | Memory loss, Mild cognitive impairment (MCI), Multiple sclerosis (MS), Myocardial infarction (MI), Neck pain, Neuropathic pain, Obstructive sleep apnea (OSA), Opioid abuse, Other demyelinating diseases, Other drug abuse, Other pain, Other substance use disorders (SUD), Overdose |
| Clinical Conditions & Comorbidities (P–V) | Parkinson's disease, Personality disorder, Pituitary disorders, Post-concussive syndrome, Post-traumatic stress disorder (PTSD), Psychosis, REM sleep behavior disorder, Schizophrenia, Sedative abuse, Seizure, Skin disease, Sleep disorder, Smoking history, Stroke, Suicidality, Tinnitus, Urinary dysfunction, Vestibular dysfunction, Vision impairment |

**Table S1:** Table of categories and individual variables used for prediction models, with all included comorbidities listed alphabetically.
