# Supplementary material for: Long‐term prediction of epilepsy following traumatic brain injury among veterans using routine clinical data
Source: Epilepsia. 2026 Aug 4;67(11):5840–50. doi: 10.1002/epi.70430 (PMC13768913; doi:10.1002/epi.70430)
Supplement: Supplementary file 3 [file EPI-67-5840-s003.docx]

|  | **Two‐year follow up, n = 105,313 (2,256 epilepsy cases)** | | | | | |
| --- | --- | --- | --- | --- | --- | --- |
| **Risk**  **mode**l | **Percentile threshold** | **N identified**  **at risk** | **SN (% of all cases)** | **FPR** | **PPV** | **Accuracy** |
| 1 | 1% | 335 | 5.5% | 0.010 | 0.11 | 0.970 |
| 2 | 2% | 639 | 10.5% | 0.018 | 0.111 | 0.963 |
| 3 | 3% | 978 | 13.7% | 0.029 | 0.095 | 0.954 |
| 4 | 4% | 1329 | 15.8% | 0.040 | 0.081 | 0.943 |
| 5 | 5% | 1663 | 19.1% | 0.050 | 0.078 | 0.934 |
| 6 | 10% | 3215 | 28.5% | 0.098 | 0.060 | 0.889 |
| 7 | 15% | 4839 | 34.9% | 0.149 | 0.049 | 0.840 |
| 8 | 20% | 6385 | 40.8% | 0.198 | 0.043 | 0.794 |
|  |  |  |  |  |  |  |
|  | **Five‐year follow up, n = 106,579 (3,522 epilepsy cases)** | | | | | |
| **Risk**  **model** | **Percentile threshold** | **N identified**  **at risk** | **SN (% of all cases)** | **FPR** | **PPV** | **Accuracy** |
| 1 | 1% | 298 | 5.0% | 0.008 | 0.178 | 0.961 |
| 2 | 2% | 609 | 9.8% | 0.016 | 0.171 | 0.954 |
| 3 | 3% | 955 | 13.2% | 0.026 | 0.147 | 0.946 |
| 4 | 4% | 1291 | 16.2% | 0.036 | 0.132 | 0.937 |
| 5 | 5% | 1611 | 18.0% | 0.046 | 0.118 | 0.928 |
| 6 | 10% | 3327 | 27.4% | 0.098 | 0.087 | 0.881 |
| 7 | 15% | 4933 | 36.7% | 0.147 | 0.079 | 0.837 |
| 8 | 20% | 6585 | 44.2% | 0.198 | 0.071 | 0.790 |
|  |  |  |  |  |  |  |
|  | **Ten‐year follow up, n = 107,987 (4,930 epilepsy cases)** | | | | | |
| **Risk**  **model** | **Percentile threshold** | **N identified**  **at risk** | **SN (% of all cases)** | **FPR** | **PPV** | **Accuracy** |
| 1 | 1% | 337 | 6.4% | 0.008 | 0.282 | 0.950 |
| 2 | 2% | 687 | 11.1% | 0.017 | 0.239 | 0.943 |
| 3 | 3% | 1025 | 14.0% | 0.026 | 0.202 | 0.935 |
| 4 | 4% | 1385 | 17.0% | 0.037 | 0.182 | 0.927 |
| 5 | 5% | 1724 | 19.9% | 0.046 | 0.171 | 0.919 |
| 6 | 10% | 3384 | 30.7% | 0.095 | 0.134 | 0.878 |
| 7 | 15% | 5017 | 39.3% | 0.143 | 0.116 | 0.835 |
| 8 | 20% | 6668 | 47.3% | 0.193 | 0.105 | 0.792 |

**Table S2:** **PTE prediction model results on test data shown for Logistic Regression.** Models were tasked to predict who would develop epilepsy at 2,5, and 10 year follow-up after TBI using only pre-injury information. Performance was similar across follow up time windows. Percentile detection thresholds were determined from training data. At 2 year follow-up from injury using risk model eight, 40.8% of all individuals who subsequently developed epilepsy in the TBI cohort were correctly identified. Conversely, risk model 2 at 2 year follow up identified just 10.5% of epilepsy cases, but with a low false positive rate of 0.018 among Veterans who did not develop epilepsy.
